# Computational Intelligence in Remote Sensing: An Editorial

**DOI:** 10.3390/s20030633

**Published:** 2020-01-23

**Authors:** Manuel Graña, Michal Wozniak, Sebastian Rios, Javier de Lope

**Affiliations:** 1 Computational Intelligence Group, University of the Basque Country, UPV/EHU, Computer Science Faculty, 00685 San Sebastian, Spain; 2 Department of Systems and Computer Networks, Wroclaw University of Science and Technology, 50-370 Wroclaw, Poland; michal.wozniak@pwr.wroc.pl; 3 Ingeniero Civil Industrial, Universidad de Chile, Santiago 0324234, Chile; srios@dii.uchile.cl; 4 Department of Artificial Intelligence, Universidad Politecnica de Madrid, 28040 Madrid, Spain; javier.delope@upm.es

**Keywords:** hyperspectral images, radar, computational intelligence, remote sensing, evolutionary computation, feature extraction, classification

## Abstract

Computational intelligence is a very active and fruitful research of artificial intelligence with a broad spectrum of applications. Remote sensing data has been a salient field of application of computational intelligence algorithms, both for the exploitation of the data and for the research/ development of new data analysis tools. In this editorial paper we provide the setting of the special issue “Computational Intelligence in Remote Sensing” and an overview of the published papers. The 11 accepted and published papers cover a wide spectrum of applications and computational tools that we try to summarize and put in perspective in this editorial paper.

## 1. Introduction

The appearance of new and more powerful remote sensing technologies has produced a surge of remote sensing data to be processed for a variety of applications in the natural sciences, such as agriculture, forestry or ecological monitoring, and others, such as the automotive industry. This special issue contains a broad sample of such applications, including indoor crowd detection and localization by means of anonymous and non invasive sensors [1]; applications in the automotive industry, such as the detection of the incoming obstacles/vehicles by on-board radar [2], and the detection of highly contaminant vehicles [3]; land cover segmentation for several purposes, such as ecological monitoring [4,5], and land uses [6,7].

The surging abundance of data has been accompanied by a blooming of computational resources and methods for analysis, such as evolutionary and random search algorithms, particle swarm optimization (PSO) [7], and ant colony optimization (ACO) [8], which are often extended as multi-objective optimization algorithms. Computational intelligence tools are increasingly being used for pre-processing, enhancement, classification, and construction of thematic maps, change detection, target detection, sub-pixel resolution analysis, and other general processes. The renaissance of artificial neural networks, with the resurgence of deep learning approaches, has injected new vitality in various research fields that deal with remote sensing data analysis, but also classical approaches such as cellular automata [6] have a renewed application value.

Of paramount importance for the development of reproducible science is the availability of data repositories and open source codes that may be used by researchers across the world to confirm or refute claimed results. The open source code has boosted many data science applications, allowing the researchers to work on high-level developments and providing a unified set of tools, and the open data initiatives promise to provide an additional boost to remote sensing research areas. Dealing with changing, non-stationary data considered in longitudinal studies [4] is a main challenge faced by the new generation of remote sensing data analysis tools. Large datasets require big data approaches to manage the complexity of dealing with the data per se, and the latest hardware resources, such as the next generation graphical processing units (GPU) [5]. Finally, data fusion techniques exploiting various data sources, such as multimodal image fusion or others, are a major challenge for the future of the field.

## 2. Special Issue Contributions

Perturbations of the atmospheric conductivity of electromagnetic waves have a very undesirable effect in civil and military communications, including the disability of specific weapon systems that rely on such wireless communication. An atmospheric duct is a layer of the atmosphere that causes electromagnetic waves to bend toward the earth’s surface, impeding wireless message transmission and the normal operation of electromagnetic weapon and communication systems. The detection and measurement of atmospheric ducts is based on the relations between radar echo and refractivity. The techniques estimate the refractivity from clutter (RFC). The capability of measuring the height at its base of the atmospheric duct trapping layers by measuring the ultrahigh frequency (UHF) signal strengths was demonstrated. This is the basis for the method of remote sensing refractivity profiles from Global Positioning System (GPS) signals, which reduces to an optimization problem. In [9], it is stated as a multi-objective optimization problem including ground-based GPS phase delay, propagation loss, effect of source frequency and receiving antenna height. Authors explore the performance in terms of diversity and convergence of solution sets for seven multi-objective evolutionary algorithms with three performance metrics: hypervolume, inverted generational distance, and the averaged Hausdorff distance, conducted an experimental comparison on three groups of test situations.

Hyperspectral sensors acquire hundreds of contiguous spectral bands for each image pixel, providing abundant spectral information about the surface of the Earth. Such spectral information abundance makes it difficult to improve sensor spatial resolution, so that mixed pixels, a mixture of more than one ground object in a single pixel, are frequent in hyperspectral images. Spectral unmixing is a rather relevant technique for hyperspectral remote sensing image analysis, decomposing the spectrum of a mixed pixel into a collection of constituent spectra (i.e., endmembers) and a set of corresponding fractions (i.e., abundances). Endmember identification/extraction is a key step in spectral unmixing. Many algorithms have been proposed to extract endmembers automatically on the basis of the linear spectral mixture model (LSMM). A line of research employs ant colony optimization (ACO) algorithms, regarding hyperspectral image endmember extraction, as a combinatorial optimization problem, despite its high computational cost. A promising path of development is the use of graphical processing units’ (GPUs) massive parallelism, however their architecture forces the redesign of the algorithm and its application in order to take full advantage of computing resources on GPUs. The special issue contribution [8] proposes a multiple sub-ant-colony-based parallel design of the ACO for endmember extraction (ACOEE), in which an innovative mechanism of a local pheromone for sub-ant-colonies enables ACOEE execution to fit in a multi-GPU system avoiding excessive load due to the synchronization among different GPUs.

In a different research line on hyperspectral image processing, dimension reduction can effectively speed up the processing of hyperspectral sensor data. In addition, it can also extract important features of hyperspectral sensor data at the same time. Thus, dimension reduction is necessary before hyperspectral image processing is performed. It is of great significance to further promote the development of the hyperspectral sensor’s application topics, such as vegetable, agriculture, oil, geology, urban, land use, water resources, and disaster [3]. The dimension reduction method can be divided into the feature extraction method and feature selection method. Band selection is the most commonly used method for the dimension reduction by feature selection on hyperspectral images. Band selection is often based on information entropy or interclass separability as evaluation criteria. In [7], inter-band correlation is considered as the guide for band aggregation, and information entropy and interclass separability are the dimension reduction evaluation criteria. They apply a multi-objective particle swarm optimization (PSO) algorithm because of its easy implementation and rapid convergence. In addition, they use game theory in order to solve potential conflicts when both information entropy and interclass separability are used to search for the optimal band combination. Experimental results reveal that the proposed method achieves global optima more easily than competing algorithms, obtaining a better band selection combination that improves ensuing classification results.

The way a remote sensing image must be segmented is directed by the user requirements, so segmentation algorithms need to be highly adaptable and are subjected to the scarcity of labeled data because of the high cost of obtaining ground truth information. The Multi-Gradient based Cellular Automaton (MGCA) proposed in [6] carries out multidimensional (often hyperspectral) image segmentation without projecting them to lower dimensional spaces. An evolutionary algorithm (ECAS-II) is used to find the optimal transition rules applied by the cellular automata to perform the image segmentation. Optimality is defined in reference to a collection of low dimensional images provided by the user to substantiate her segmentation requirements. This process avoids the costly gathering of ground truth data acquired in the field, as it can be carried out online on the basis of a parametrization of the desired segmentation extracted from a set of examples. The segmentation process can be followed by a classification step, which can be realized by of-the-shelf classifier systems, such as support vector machines (SVM). The conjunction of all of these steps leads to a workflow that allows obtaining hyperspectral image segmenters using very small training sets.

The study of time series of remote sensing data allows to detect changes in the land cover that provide insights into the actual environmental processes going on, such as the assessment of the climate change impact on land usage. Monitoring long-term trends of green vegetation in a semi-arid region gives valuable insight into dependencies and changing quantities influenced by climate variability. The detection of trends in the changes of green vegetation over time is essential for the assessment of the impacts of climate variability on the Land Use Land Cover Change (LULCC) of a region. For the monitoring and analysis of green vegetation, the infrared (IR) and near infrared (NIR) spectral channels are best suited, since they discriminate between green and active vegetation versus woody vegetation or organic litter. Fractional cover (FCover) data is a derived product out of Landsat imagery and shows the fractions of existing land cover in one pixel as percentages that are contained within the pixel. A two step method for employing satellite data to evaluate spatial and temporal patterns of environmental indices of interest is presented in [4]. In the first step, linear regression coefficients are extracted for each area in the image. These coefficients are then employed as a response variable in a boosted regression tree with geographic coordinates as explanatory variables. The method is demonstrated on a substantive case study comprising 30 years of satellite derived fractional green vegetation cover for a large region, including separate analyses over sub-regions of the study region. All together, eight scenarios have been investigated, namely the whole data set covering 30 years, then three data sets covering a decade each, then, the four quadrants of the image over all years.

Accurate image registration is often a required preprocessing before the subsequent processing of remote sensing images to obtain some product. Image registration based on phase correlation has attracted great attention due to its high accuracy and efficiency. However, the Discrete Fourier Transform (DFT) of a discretized image is periodic. In practical applications, it is highly unlikely that opposite borders of an image are similar, hence DFT-processed images always show strong discontinuities across the frame border. The discontinuities cause a severe artifact in the Fourier Transform, namely the known cross structure composed of high energy coefficients along the axes. This image border effect corrupts registration processes lowering accuracy and success rate. Conventional approaches dealing with this artifact rely on low pass filtering of the image, which introduces further artifacts. In [10], another way of tackling with the image border artifact is proposed, namely decomposing the image into two images: one the periodic image and the other the smoothed image. Replacing the original image by the periodic one does not suffer from the effect on the image border when applying Fourier Transform. The smooth image is analogous to an error image, which has little information except at the border. Extensive experiments show that the novel algorithm of eliminating the image border can improve the success rate and accuracy of phase correlation based image registration.

In recent years, radar systems have been installed in automobiles to detect targets located in multiple directions. Typically, automotive radar systems use frequencies in the 24-GHz or 77–81-GHz band. Since such a high frequency band is used, the miniaturization of the radar antenna system has become possible. In the automotive radar system, the number of receiving antenna elements is gradually decreasing to reduce the manufacturing cost of the radar. Therefore, array interpolation techniques must be used for direction of arrival (DOA) estimation to achieve high angular resolution, often applying linear least squares (LLS) method. However, a side effect of LLS methods is that the amplitudes of the interpolated array elements may not be in the same range of the original array elements. In addition, the phases of the interpolated array elements are not precisely generated. The special issue contribution [2], proposes an array transformation matrix able to generate accurate phases for interpolated array elements improving DOA estimation performance, while preserving the amplitudes of the array elements. Additionally, they provide an innovative power calibration method for interpolated received signals. Simulations confirm that the array interpolation accuracy and DOA estimation performance of the proposed method improve those of the conventional methods. Moreover, the method is validated using data obtained from a commercial radar system.

Nowadays, digital data exchanged through communication networks are exposed to various forms of malicious attacks. A small piece of additional information, i.e., a watermark, can be embedded within the data to identify their origin and verify that it has not been corrupted in any way. The watermark can be embedded in the spatial or frequency domains, where different transformations are applied, such as, for example, the discrete cosine or discrete wavelet transformations. In the latter case, some coefficients of the transformed data are changed to introduce the watermark. With the wide accessibility of 3D scanning devices, a huge amount of discrete points (i.e., point clouds), acquired from the surfaces of 3D objects, are currently obtained easily. Most 3D point cloud watermarking techniques apply Principal Component Analysis (PCA) to protect the watermark against affine transformation attacks. Unfortunately, they fail in the case of cropping and random point removal attacks. In [11], an alternative approach is proposed that solves these issues efficiently. A 3D convex hull point cloud registration technique is developed. The scale and the initial rigid affine transformation between the watermarked and the original point cloud can be estimated in this way to obtain a coarse point cloud registration. An iterative closest point algorithm is performed after that to align the attacked watermarked point cloud to the original one completely. The watermark can then be extracted from the watermarked point cloud easily. The extensive experiments confirmed that the proposed approach resists the affine transformation, cropping, random point removal, and various combinations of these attacks.

Remote sensing big data is generally characterized by huge volumes, diversity, and high dimensionality. Mining hidden information imposes significant computational challenges. Clustering is an important data mining technique widely used in processing and analyzing remote sensing imagery. However, conventional clustering algorithms are designed for relatively small datasets. When applied to remote sensing data they are, in general, too slow or inefficient for practical use. In this special issue, authors of [5] propose a parallel subsampling-based clustering method for improving the performance of remote sensing big data clustering in terms of both efficiency and accuracy, leveraging a novel subsampling-based data partitioning method to realize three-step parallel clustering, effectively solving the notable performance bottleneck of the existing parallel clustering algorithms; that is, they must cope with numerous repeated calculations to get a reasonable result. Furthermore, authors propose a centroid filtering algorithm removing subsampling errors. Their proposal has been implemented on a Hadoop platform by using the MapReduce parallel model. Experiments conducted on massive remote sensing imageries showed (1) improved accuracy over conventional remote sensing clustering algorithms when handling larger image data, (2) computational scalability achieved with increased computing nodes added, and (3) much less real time spent compared to existing parallel clustering algorithm for remote sensing data.

Indoor crowd localization and counting in big public buildings pose problems of infrastructure deployment, signal processing, and privacy. Conventional approaches based on optical cameras, either in the visible or infrared range, received signal strength in wireless networks, sound or chemical sensing in sensor networks need careful calibration, noise removal, and sophisticated data processing to achieve results in limited scenarios. Moreover, personal data protection is a growing concern, so that detection methods that preserve the privacy of people are highly desirable. The aim of [1] is to provide a technique that may generate estimations of the localization of people in a big public building using anonymous data from already-deployed Wi-Fi infrastructure. We present a method applying geostatistical techniques to the access data acquired from access points in an open Wi-Fi network. Specifically, only the time series of the number of accesses *per* access point is required. Geostatistical methods produce a 3D high-quality spatial distribution representation of the people inside the building on the basis of the interaction of their mobile devices with the access point.
